# Women’s expectations and experiences of labor induction – a questionnaire-based analysis of a randomized controlled trial

**DOI:** 10.1186/s12884-021-03786-6

**Published:** 2021-05-04

**Authors:** Moa Strandberg, Tove Wallstrom, Eva Wiberg-Itzel

**Affiliations:** 1 Department of Clinical Science and Education Karolinska Institute, Sodersjukhuset, 118 83 Stockholm, Sweden; 2 Department of Obstetrics and Gynecology, Sodersjukhuset, 118 83 Stockholm, Sweden

**Keywords:** Psycho-emotional aspects of childbirth, Labor induction, Misoprostol

## Abstract

**Background:**

Although labor induction is a commonly used procedure in obstetrical care, there are limited data on its psycho-emotional effects on the woman. This study analysed the expectations and experiences of women in different routes of labor induction. The study’s primary aim was to compare women’s delivery experience if induced by orally administrated misoprostol (OMS) compared with misoprostol vaginal insert (MVI). Secondly, an evaluation of women’s general satisfaction with induced labor was made, and factors associated with a negative experience.

**Methods:**

Primiparous women (*n* = 196) with a singleton fetus in cephalic presentation, ≥ 37 weeks of gestation, with a Bishop’s score ≤ 4 planning labor induction were randomly allocated to receive either OMS (Cytotec®) or MVI (Misodel®). Data were collected by validated questionnaires, the Wijma Delivery Expectation/Experience Questionnaire (A + B). The pre-labor part of the survey (W-DEQ version A) was given to participants to complete within 1 hour before the start of induction, and the post-labor part of the questionnaire (W-DEQ version B) was administered after birth and collected before the women were discharged from hospital.

**Results:**

It was found that 11.8% (17/143) reported a severe fear of childbirth (W-DEQ A score ≥ 85). Before the induction, women with extreme fear had 3.7 times increased risk of experiencing labor induction negatively (OR 3.7 [95% CI, 1.04–13.41]).

**Conclusion:**

No difference was identified between OMS and MVI when delivery experience among women induced to labor was analysed. Severe fear of childbirth before labor was a risk factor for a negative experience of labor induction.

**Trial registration:**

Clinical trial register number NCT02918110. Date of registration on May 31, 2016.

**Supplementary Information:**

The online version contains supplementary material available at 10.1186/s12884-021-03786-6.

## Background

The induction of labor is a commonly used obstetrical intervention in low-income as well as developed countries. This procedure is usually performed late in pregnancy to prevent maternal or fetal complications. During the past 5 years, 17% of all singleton pregnancies in Sweden were induced [1]. Most research regarding labor induction has focused on various methods’ efficiency and safety, with little attention paid to women’s experiences and preferences [2–5].

Compared with the spontaneous onset of labor, induction may increase the woman’s risk of a less positive birth experience [6, 7]. A woman’s level of satisfaction with her first childbirth has immediate and long-term effects on her health and relationship with the infant. Dissatisfaction increases the risk of post-traumatic stress disorder (PTSD) and possible preference for CS with her next pregnancy [8, 9].

Methods of induction include amniotomy, mechanical dilation with a balloon catheter, pharmacological induction with prostaglandin E1 (misoprostol, Cytotec®), or prostaglandin E2 (dinoprostone as Propess® or Minprostin®). Misoprostol has been found to stimulate uterine contractions and cervical ripening effectively. In 2011 the World Health Organization (WHO) listed misoprostol as an essential medicine for labor induction [10]. The optimal route of administration of misoprostol is controversial. It can be produced in various formulations such as vaginal, rectal, sublingual, and oral, as a tablet or solution. To date, no study has compared orally administered misoprostol as a solution (Cytotec® Pfizer, NY, USA) with the vaginal insert of misoprostol (Misodel® Ferring, Malmo, Sweden) regarding efficiency and safety. Both formulations have recently been shown to be active and safe when used to induce labor in primiparous women [11–13]. However, none of the studies have investigated the women’s psycho-emotional experiences of labor induction via these methods.

## Methods

### Aims of the study

The study’s primary aim was to compare women’s delivery experience if they were induced by orally administrated misoprostol (OMS) compared with misoprostol vaginal insert (MVI); secondly, an evaluation of women’s general satisfaction with induced labor was made, and factors associated with a negative experience.

### Ethical approval

The regional ethics committee approved the study (Uppsala, file record: 2016/047 and the National Medical Product Agency (Eudura CT-2016-000949-31) in 20,160,713), and the study was also registered by the Clinical Trial (clinical trial register number NCT02918110). The study complies with the World Medical Association Helsinki Declaration regarding the ethical conduct of research involving human subjects. The study protocol was developed following the consort guidelines for clinical trials. Written informed consent was obtained from all the women before inclusion in the study.

### Study design

This open label randomized controlled trial of Misodel vs. Cytotec was performed at a secondary referral hospital, Soder Hospital, in Stockholm, Sweden. It was conducted following the CONSORT guidelines. The study was performed during the period of October 1, 2016, to February 21, 2018. As a sub-study of the RCT study, a questionnaire-based survey was conducted where orally administered misoprostol (OMS) was compared to the vaginal insert of misoprostol (MVI) on general maternal satisfaction.

In this sub-analysis, data from the questionnaires (Wijma Delivery Expectation/Experience Questionnaire A + B*)* assessing the participant’s expectations and induction experiences is presented. The W-DEQ A + B measures the woman’s experience of childbirth before and after delivery. The questionnaires include 33 items on a 6-point Likert scale, each scoring from 0 (not at all) to 5 (exceedingly fear). The sum score ranges from 0 to 165, the higher the score, the worse the woman’s experience.

### Study population

The inclusion criteria were primiparous women with a viable singleton fetus in cephalic presentation, ≥ 37 weeks of gestation, and a Bishop’s score ≤ 4.

The exclusion criteria were previous uterine surgery, prenatal fetal complications such as severe intrauterine growth restriction (IUGR), or an abnormal fetal heart rate pattern on the CTG (cardiotocography) after arrival to the hospital. Additionally, women unable to understand the questionnaire written in Swedish were excluded.

Women matching the inclusion criteria received verbal and written information about the study. Information was provided by an obstetrician or a midwife in clinical service when the woman arrived for elective induction. After maternal written consent was given, randomization was performed using the sealed envelopes. The envelopes were opened, and all participants were then asked to complete the validated pre-labor questionnaire (Wijma Delivery Expectation Questionnaire, W-DEQ version A) before the medicine was administered. The survey measures a woman’s prenatal perception and expectation of childbirth. Higher total scores indicate a greater fear of childbirth [14]. During induction and delivery, the personnel involved had no information about the women’s W-DEQ A score.

If the woman was randomized to receive OMS, the induction was carried out according to the clinic’s routine labor protocol. 2.5 ml of a titrated oral misoprostol solution (10 μg/ml), which could be repeated every second hour with a maximum of eight doses (= 200 μg/24 h), would be given. The exact dosage in the solution was achieved using a misoprostol tablet (200 μg) pulverized and dissolved in 20 ml water. The Swedish Institute of Pharmacology has tested this administration method and approved it to be accurate in correct dosages [15]. If the woman experienced painful contractions at the next dose, the induction was paused for 1 hour while waiting for the contractions spontaneously. If the contractions disappeared, the woman received another dose, but a digital vaginal examination was performed to evaluate cervical ripening if the contractions continued spontaneously. When BS was ≥6, before or after the eight doses, amniotomy and oxytocin were used to augment uterine contractions if necessary.

If the woman was randomized to MVI, the vaginal tablet was placed high in the posterior vaginal fornix at the start of the induction. The tablet was placed in a reservoir that could easily be removed at any time. Due to the slow-release profile (7 μg/hour), the reservoir could be left 24 h (158 μg/24 h). Fetal monitoring with CTG was performed every 4–6 h or when regular contractions were established. The MVI was removed when labor was established or if the 24 h dosing period was complete.

If further ripening of the cervix was needed, a balloon catheter (Bard®) or 1–2 mg vaginal insert of prostaglandin E2 dinoprostone (Minprostin®) was used. Active labor was handled according to the clinic’s standard delivery ward protocols. For pain relief during induction and delivery, paracetamol, a short-acting opioid, nitric oxide inhalation, epidural analgesia, and paracervical block were offered. All participants remained in the delivery ward throughout induction.

After delivery, the participants were asked to complete the validated post-labor questionnaire (Wijma Delivery Experience Questionnaire, W-DEQ version B) before discharge from the hospital. The survey measures the woman’s experience of childbirth, and like the pre-labor questionnaire, the W-DEQ B consists of 33 items, each scored from 0 to 5. A high total score indicates a negative experience of childbirth [14]. Background characteristics and delivery outcomes were collected from the maternal medical files. All personal data were encoded, so individuals could not be identified in the analysis.

### Statistics

The primary analytical approach of this RCT was Intention to treat. Normally distributed continuous data are shown as means with standard deviations and compared by one-way ANOVA or independent t-test. For analysis of the categorical variables such as mode of delivery, the chi-square test was used*.* The cut-off between good/moderate and negative experience of childbirth (W-DEQ version B) was set at a sum score of ≥66, which was the 75th percentile in the original study of childbirth experience [15]. According to the questionnaire guidelines, missing internal data could be replaced by the group mean for an unanswered question. Consistent with several other studies in the field [16–18], the cut-off level for severe fear of childbirth was set to a total score of ≥85 (W-DEQ version A).

Logistic regression was used to study the association between a negative experience of induced labor and each of the independent factors: maternal age, method of induction, Bishop’s score (BS), time of delivery, mode of delivery, low Apgar score, anal sphincter injury and low pH in arterial cord blood at delivery. Our model strategy was as follows: first, unadjusted associations with each factor were studied; second, the adjusted association concerning the risk factors measured was studied in a multivariable model with all factors included. Statistical analyses were performed using SPSS version 20.0 (SPSS Inc., Chicago, IL.). *P*-values < 0.05 were regarded as statistically significant.

The power calculation was based on the primary study of the efficiency of the two different treatments investigated in the randomized trial.

## Results

**Fig. 1:**
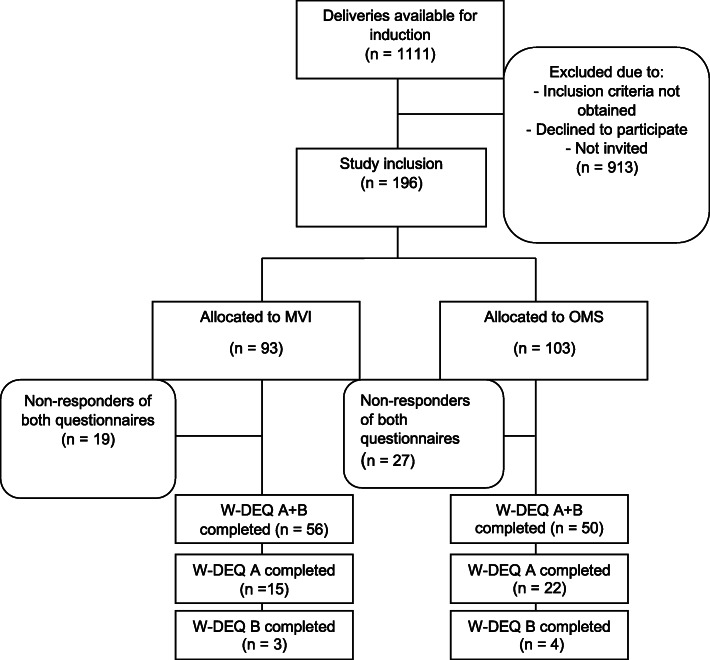
Flowchart of the participants

One hundred ninety-six primiparous women were included in the study; 93 were allocated to MVI and 103 to OMS as the method of induction (Fig. 1). The results of this questionary-based study were presented in three groups according to the result of the W-DEQ B. One group with W-EDQ B < 66p (*n* = 65), one group with W-EDQ B > =66p (*n* = 41), and one group of non-responders of the questionnaire (*n* = 83). No differences were shown in background characteristics among the three groups (Table 1).

**Table 1:**
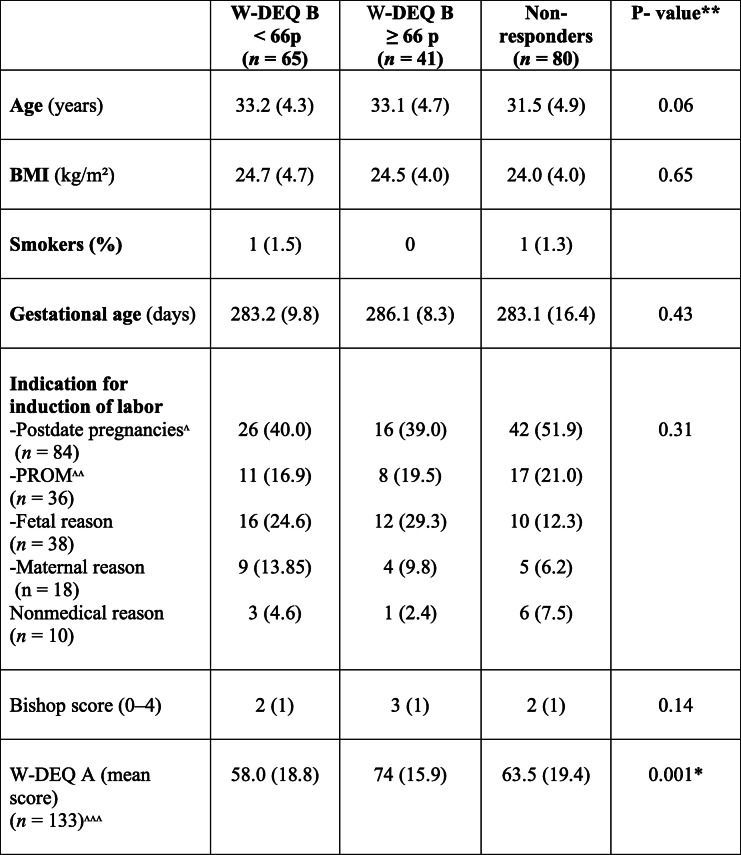
Background characteristics of the women included in the project. Data presented per group (low/high, or non-responders) according to W-DEQ B*questionnaire. Values are numbers (%) or mean (SD). *N* = 186

* Wijma Delivery Experience Questionnaire, W-DEQ

** *P* values < 0.05 were considered statistically significant, ^ > 41 weeks, ^^ Pre -rupture of the membranes, ^^^ only five women complete the W-DEQ B

Among participating women, 143 (73%) returned the pre-labor questionnaire (W-DEQ A), and 113 (57%) returned the post-labor questionnaire (W-DEQ B). Both surveys were completed by 106 (54.1%) women (Fig. 1). All the pre-labor surveys and 75% (86/113) of the post-labor questionnaires were completed before the women were discharged from the hospital. The ‘late-responders’ (*n* = 27) were equally distributed between the OMS and MVI groups and did not differ related to W-DEQ B’s mean value.

A comparison between the two different forms of induction OAS and MVI and the questionnaires’ results were made. No significant difference in the questionnaire score was found among the various forms of the inductions (W-DEQ A; *p* = 0.45 and W-DEQ B; *p* = 0.50).

W-DEQ B’s mean score among all the women in the study was 61.2p (SD:22.9), and 39% (44/113) reported a W-DEQ B score ≥ 66p, corresponding to a higher level of negative experience in induction and childbirth. The mean score of the pre-labor questionnaire W-DEQ A differed between women with or without high scores of W-DEQ B. Women with W-DEQ B > =66p had the highest mean score of W-DEQ A; 74p (SD 15.9). Women with W-DEQ < 66p had the lowest means score; 58p (SD 18.8) (Table 1).

Labor outcomes are presented in Table 2. Among the included women, 62.2% (130/196) were delivered vaginally without instruments; 16.8% were delivered by CS (33/186), and 16.8% (33/186) by vacuum extraction. A significantly higher frequency of spontaneous vaginal deliveries was found in the W-DEQ B ≤ 66 group, 78.3% vs. 63.4 and 53.6% (*p* = 0.03). Mean delivery time (from start of induction to delivery) was comparable between the three groups (22.6 h vs. 24.3 h vs. 23.1 h, *p* = 0.71) as well as the use of EDA (epidural anaesthesia) (78.3 vs. 90.9 vs. 88%, *p* = 0.20) and oxytocin (66.7 vs. 70.5 vs. 78.3%, *p* = 0.34). Fetal delivery outcomes are presented in Table 3. No differences between the groups were observed.

**Table 2:**
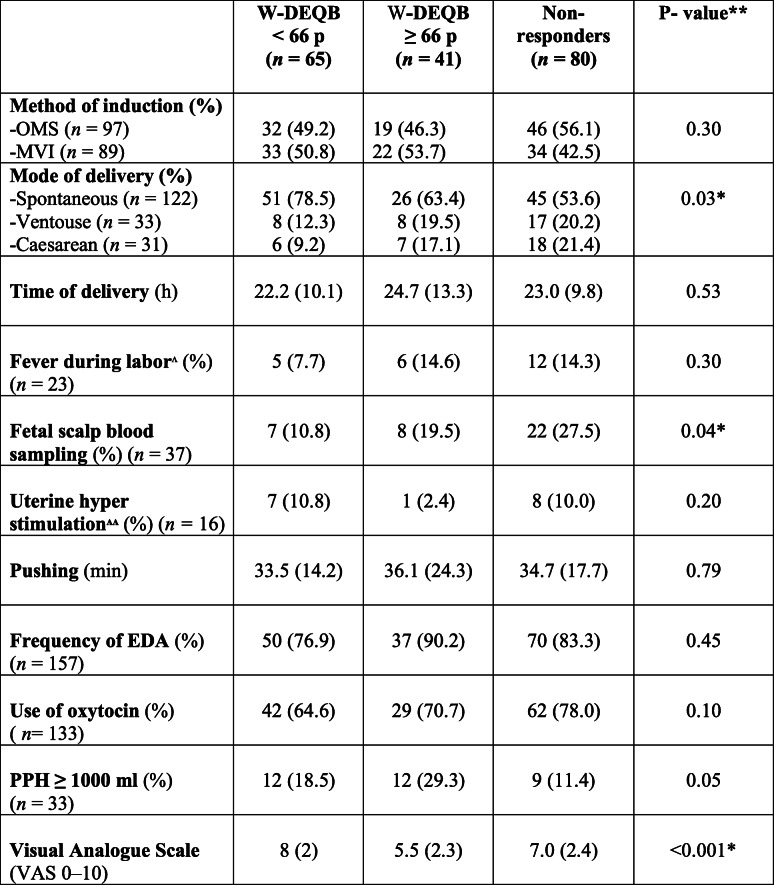
Delivery outcomes presented per group (low/high, or non-responders) according to W-DEQ B* questionnaire. Data are presented as numbers (%) or mean (SD) *N* = 186

* Wijma Delivery Experience Questionnaire, W-DEQ, ** P values < 0.05 were considered significant, ^ Fever defined as body temperature > 37.5°, ^^ > 5 contractions/10 minutes with affected fetal heart rate registered on the CTG

**Table 3:**
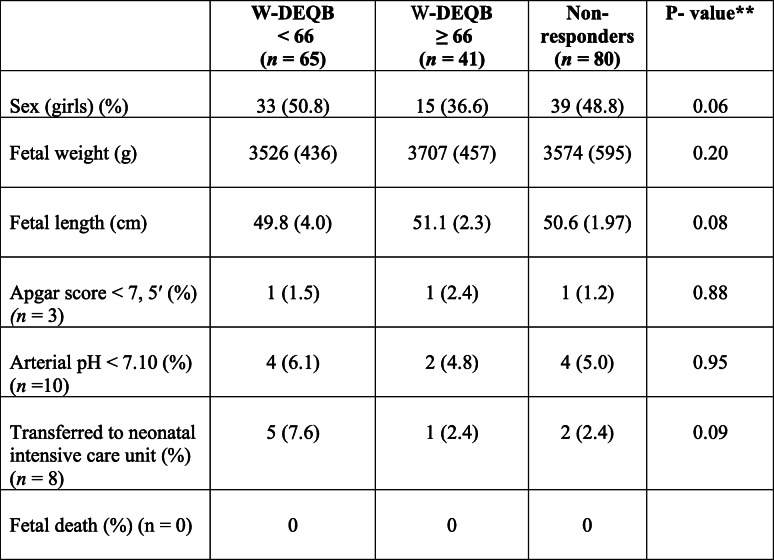
Delivery outcomes of the newborns presented per group (low/high, or nonresponders) according to W-DEQ B questionnaire*. Values are numbers (%) or mean (SD). *N* = 186

*Wijma Delivery Experience Questionnaire, W-DEQ ** *P* values<0.05 were considered statistically significant

The mean score of the pre-labor questionnaire W-DEQ version A was 64.1p (SD19.1), with 11.8% (17/143) women reporting a high score, associate with severe fear of childbirth, before induction was started (cut-off score ≥ 85p, used in earlier publications).

Logistic regression was made and showed no association between the maternal age, Bishop Score, the medicine used for induction (OMS or MVI), meantime of labor, Apgar score at 5 min, or pH in cord blood < 7.10, and satisfaction of induction (Table 4). Women with a high W-DEQ A score before the start of induction had a 3.7 increased risk of reporting a negative labor experience independent of whether OMS or MVI had been used (OR 3.7; 95%, CI 1.04–13.41).

**Table 4:**
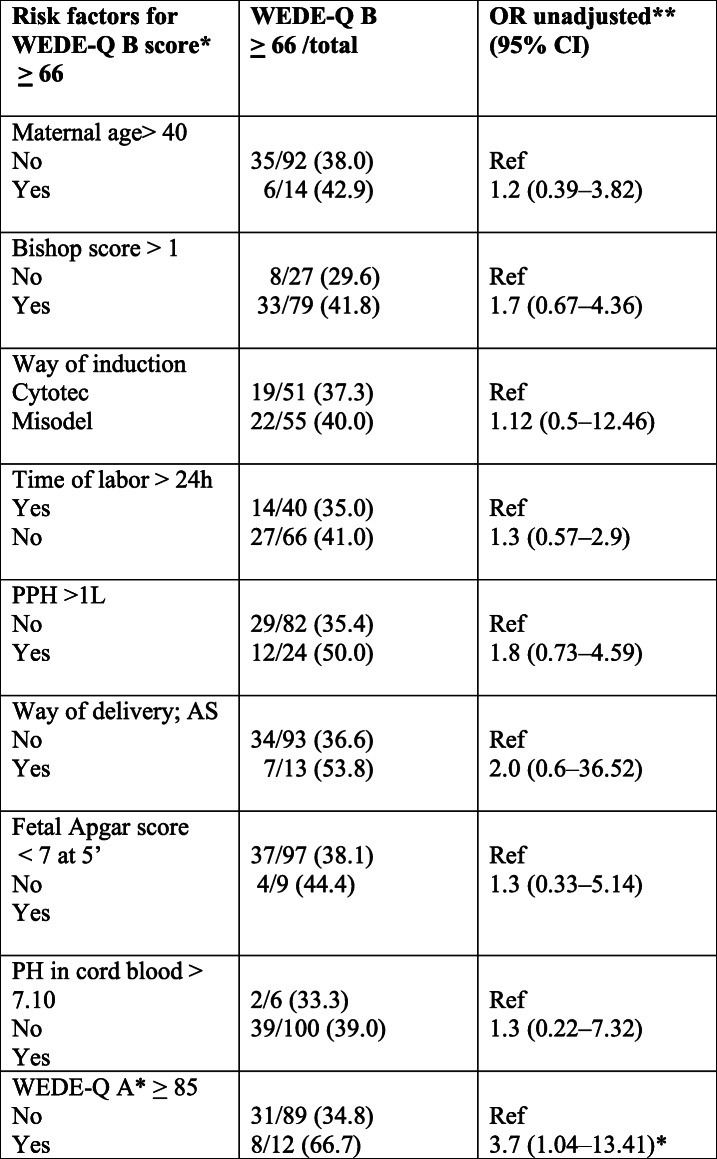
Associations between possible risk factors and the risk of bad labour experience. Values are expressed as odds ratio (OR) with corresponding 95% confidence intervals (CI). (*n* = 106)

*Wijma Delivery Experience Questionnaire, W-DEQ

**P values<0.05 were considered statistically significant

## Discussion

This questionnaire-based study’s primary finding was that no difference in terms of satisfaction was shown between the two different induction methods used in this study. The women were equally satisfied or dissatisfied.

Women presented with a high score on the questionnaire W-DEQ A strongly correlated to a less satisfactory induced labor experience. A high score on the questionnaire W-DEQ A before the start of induction was reported by 11.8% of participating women. Further, a correlation between the results of W-DEQ A och B was shown. Women whit high W-DEQ A levels before labor also presented high levels of W-DEQ B after delivery. They also had the lowest frequency of spontaneous vaginal delivery.

A legitimate question to ask is whether W-DEQ A + B is the right questionnaire to use in this context. In 2018, a Review was done by Y. Richen et al. [19] conducted on any questionnaire’s usefulness in evaluating a woman’s childbirth experience. W-DEQ’s criticism is that it is very long and challenging to fill in for the woman who will answer it. The conducted review results showed that The FOBS is likely to be a more versatile tool used in clinical practice. To this now presented work’s defence of W-DEQ A + B’s use, W-DEQ has been used by our research group in similar works in the past, which means that there is an experience in interpreting the values.

Psycho-emotional satisfaction with labor is multidimensional and necessitates the use of various measuring instruments to be estimated. Besides, data from questionnaires are interpreted differently, and the results of this study may be difficult to compare. Brane et al. used the same instrument to compare women’s induced labor experiences to those who had a spontaneous onset of labor. The W-DEQ version B’s mean score was 68 in both groups of included women [20]. In the current study, the W-DEQ B questionnaire’s mean score was 61.2 (SD 22.9), which might indicate a slightly more positive delivery experience. Besides, Ulfsdottir et al. used the same instrument to assess delivery experiences among 446 Swedish primiparous women with spontaneous labor onset. In 44% of women, a mostly negative labor experience was reported (W-DEQ B ≥ 66) [21].

Consistent with the proportion reporting a high score in this study (11.8%), more than one-tenth (10–15%) of primiparous women in a European multi-centre study reported a severe fear of childbirth, defined by the sum score on the W-DEQ A ≥ 85 [17, 18]. Extreme fear of childbirth has been correlated to prolonged labor [22]. Interestingly, such a correlation could not be confirmed in this study. Alehagen has also reported an association between severe fear before and after birth at al. in 2006 [23].

### Strengths and limitations

To our knowledge, this is the first evaluation that compared women’s experiences with OMS to MVI for the induction of labor. Also, it is one of the few recent studies to investigate women’s expectations and experiences of induced labor. This study was performed in one of Sweden’s largest delivery wards, with approximately 7900 deliveries per year. The choice of the Wijma Delivery Expectation/Experience Questionnaire as an instrument will enable results from future studies to be compared with the present trial, as the questionnaire has been translated and validated in numerous languages (i.e., Turkish, Japanese, Italian, and Farsi) after it was initially developed in Sweden in 1998 [14].

Of the women asked to complete the questionnaire, only 57% finished the post-labor survey. During the study period, delivery care in the Stockholm region was undergoing structural changes, and the number of deliveries increased rapidly in the department. It is possible occupational stress among the personnel led to an inability to receive and remember information about the study protocol. This contributed to the high number of non-responders. It is also probable that completing a survey just after childbirth (especially a traumatic birth) is of low priority for women. Despite the busy working environment, 73% returned a completed pre-labor questionnaire. The optimal time for completion of the survey can also be discussed. It is possible women only 1 day after delivery is overwhelmed by having a healthy baby, and negative feelings from the delivery may only emerge later. However, there were no differences in satisfaction between early-responders and late-responders in this study.

Currently, neonatal, and maternal delivery outcomes in developed countries are such good; women often evaluate childbirth in psycho-emotional rather than medical terms. When counselling women before induction, we suggest attention should be paid to the woman’s expectations. Identifying individuals with a severe fear of childbirth might, with appropriate interventions during induction and delivery, potentially increase the number of satisfied women. A validated instrument for measuring women’s induced labor experiences would facilitate investigations aiming to find effective interventions.

## Conclusions

No difference was identified between OMS and MVI when delivery experience among women induced to labor was analysed. Severe fear of childbirth before labor was a risk factor for a negative experience of labor induction.

## Supplementary Information

**Additional file 1.** Wijma Delivery Expectancy/Experience Questionnaire (W-DEQ version B)

## Data Availability

Shown on request. The complete database is not publically available, but it available on request with the corresponding author after permission of The Karolinska Institute.

## Abbreviations

OMS: Orally administrated misoprostol
MVI: Misoprostol vaginal insert
W-DEQ: Wijma Delivery Expectation/Experience Questionnaire
PPH: Postpartum hemorrhage
CS: Cesarean section
PTSD: Post-traumatic stress disorder
WHO: World Health Organization
CTG: Cardiotocography
IUGR: Intrauterine growth restriction
BS: Bishop’s score
