# Supplementary material for: Women’s expectations and experiences of labor induction – a questionnaire-based analysis of a randomized controlled trial
Source: BMC Pregnancy Childbirth. 2021 May 4;21:355. doi: 10.1186/s12884-021-03786-6 (PMC8097967; doi:10.1186/s12884-021-03786-6)
Supplement: Supplementary file 1 — Additional file 1. Wijma Delivery Expectancy/Experience Questionnaire (W-DEQ version B) [file 12884_2021_3786_MOESM1_ESM.doc]

date ...........………… Code no____________

**Wijma Delivery Expectancy/Experience Questionnaire (W-DEQ version B)**

**Instructions**

This form is about different emotions and thoughts women can have afterwards about childbirth.

The answer to each question is constructed as a scale from 0 to 5. The outer ends of the scale (0 and 5 respectively) corresponds to the opposite extremes of a certain feeling or thought.

You answer each question by putting a ring around the number that best corresponds to how you now think your birth was. Answer as you now think your birth was - not as you wish it would have been!

Note that the answers are formulated so that sometimes "very" means something very positive and sometimes "much" means something very negative. Therefore, for every question you need to think about where to put your ring!

***When you have filled in the form, you are welcome to check that you have not forgotten any question***

**I How did you experience your childbirth as a whole?**

1 Extremely fantastic 0 1 2 3 4 5 Not at all fantastic

2 Extremely horrible 0 1 2 3 4 5 Not at all horrible

**II How did you feel in general during childbirth?**

3 Extremely alone 0 1 2 3 4 5 Not alone at all

4 Extremely strong 0 1 2 3 4 5 Not at all strong

5 Extremely safe 0 1 2 3 4 5 Not at all safe

6 Extremely afraid 0 1 2 3 4 5 Not at all afraid

7 Extremely extradited 0 1 2 3 4 5 Not at all extradited

8 Extremely weak 0 1 2 3 4 5 Not at all weak

9 Extremely safe 0 1 2 3 4 5 Not safe at all

10 Extremely independent 0 1 2 3 4 5 Not at all independent

11 Extremely exhausted 0 1 2 3 4 5 Not at all exhausted

12 Extremely tense 0 1 2 3 4 5 Not tense at all

13 Extremely joyful 0 1 2 3 4 5 Not joyful at all

14 Extremely proud 0 1 2 3 4 5 Not proud at all

15 Extremely abandoned 0 1 2 3 4 5 Not at all abandoned

16 Extremely collected 0 1 2 3 4 5 Not at all collected

17 Extremely relaxed 0 1 2 3 4 5 Not at all relaxed

18 Extremely happy 0 1 2 3 4 5 Not happy at all

**III What did you feel during labor?**

19 Incredible panic 0 1 2 3 4 5 Not at all panic

20 Incredible hopelessness 0 1 2 3 4 5 Not at all hopless

21 Incredible longing

for the baby 0 1 2 3 4 5 Not at all longing for the baby

22 Incredible self-confident 0 1 2 3 4 5 No self-confidence at all

23 Total trust 0 1 2 3 4 5 No trust at all

24 Incredible pain 0 1 2 3 4 5 No pain at all

**IV What happened when labor was the most intense?**

25 I behaved badly 0 1 2 3 4 5 I did not behave bad at all

26 I dared to give myself up to

what happened in my body 0 1 2 3 4 5 I did not dare to give myself up to what happened in my body

27 I totally lost my self- 0 1 2 3 4 5 I did not lose my self-control

control

**V How did you feel about giving birth to the baby?**

28 Extremely fun 0 1 2 3 4 5 Not fun at all

29 Extremely natural 0 1 2 3 4 5 Not natural at all

30 Extremely obvious 0 1 2 3 4 5 Not obvious at all

31 Extremely dangerous 0 1 2 3 4 5 Not dangerous at all

**VI Did you have thoughts during childbirth such as….**

32 ... thinking that it could happen that the baby may die during labour

Never 0 1 2 3 4 5 Very often

33 ... thinking that it could happen that the baby may got hurt during labour

Never 0 1 2 3 4 5 Very often
